# Pre-Hospital Rate-Pressure Product Is Not Positively Associated with Hematoma Expansion or Initial Hematoma Volume in Spontaneous Intracranial Hemorrhage

**DOI:** 10.3390/neurolint18010020

**Published:** 2026-01-20

**Authors:** Stephanie Q. Liang, Daniel M. Oh, Fawaz Philip Tarzi, Nerses Sanossian, David S. Liebeskind, Jeffery L. Saver, Melissa Wilson, Roy A. Poblete

**Affiliations:** 1 Department of Neurology, Keck Medical Center of University of Southern California, Los Angeles, CA 90033, USA; 2 Los Angeles General Medical Center, Los Angeles Department of Health Services, Los Angeles, CA 90033, USA; 3 Department of Neurology, Kaiser Permanente, Riverside, CA 92505, USA; 4 Department of Emergency Medicine, University of California, Los Angeles, CA 90095, USA; 5 Department of Population and Public Health Sciences, Keck School of Medicine, University of Southern California, Los Angeles, CA 90032, USA

**Keywords:** intracerebral hemorrhage, rate-pressure product, hematoma expansion, hypertension, outcomes

## Abstract

**Background**: The management of spontaneous intracerebral hemorrhage (ICH) has centered around controlling blood pressure in order to prevent hematoma expansion (HE). Rate-pressure product (RPP) has emerged as a hemodynamic marker that accounts for heart rate (HR) and systolic blood pressure (SBP), both of which are crucial in modifying shear stress to the vasculature. We hypothesized that RPP in the pre-hospital hyperacute phase is positively associated with initial hematoma volume and HE. **Methods**: We analyzed 263 patients with primary ICH from the Field Administration of Stroke Therapy-Magnesium (FAST-MAG) study with initial and interval neuroimaging. RPP was calculated as the product of HR and SBP in pre-hospital and pre-treatment phases, stratified into quintiles. HE was defined by volume expansion of >6 mL or >33% from baseline volume on repeat neuroimaging performed within 48 h of the first scan. The primary outcome was the initial hematoma volume by quintiles of hyperacute RPP. The secondary outcome was the occurrence of HE across RPP quintiles. Multivariable logistic regression was used to assess the degree to which RPP affects HE. **Results**: Of the 263 patients analyzed, 116 (44%) had HE. The proportion of patients with HE or the initial hematoma volume was not statistically significant across RPP quintiles overall. HE was significantly more common in female patients or patients on anticoagulation. **Conclusions**: Elevated RPP was not associated with increased initial hematoma volume or subsequent HE in the hyperacute period after spontaneous ICH. Future research is necessary to determine the clinical importance of RPP as a biomarker in the clinical outcome of ICH.

## 1. Introduction

Spontaneous intracerebral hemorrhage (ICH) is the second most common stroke type and is associated with a high morbidity and fatality rate [1]. An older age at event onset and worse functional outcomes are observed in ICH patients as compared to ischemic stroke, resulting in a large burden of disease [2,3]. Despite this, evidence-based medical therapies for ICH remain limited and are largely focused on preventing hematoma expansion (HE). It is generally postulated that early hematoma stabilization in the acute period by blood pressure (BP) control and hemostasis can reduce morbidity and mortality that occurs secondary to HE.

Systolic blood pressure (SBP) is implicated as a predisposing factor for both ICH onset and subsequent HE [4]. In addition to chronic hypertension, acute elevation in SBP is attributed to sympathetic hyperactivity and physiologic autoregulation in response to tissue ischemia and acute brain injury [5,6]. Although there is conflicting scientific evidence for more intensive BP lowering to improve outcomes, the common clinical practice of targeting an SBP goal of <140 mmHg is founded on its safety demonstrated in randomized, controlled trials that include the second and third Intensive Blood Pressure Reduction in Acute Cerebral Hemorrhage Trials (INTERACT2 and INTERACT3) and the Antihypertensive Treatment of Acute Cerebral Hemorrhage (ATACH)−2 trial [7,8].

The effect of heart rate (HR) with concurrent hypertension on hematoma characteristics has not been rigorously studied. Rate-pressure product (RPP) is defined as HR x SBP and represents an objective hemodynamic index that is easily calculated. RPP has been used in patients with cardiac disease, primarily as a marker of myocardial workload [9,10,11]. Similarly, in acute aortic dissection, controlling both systolic hypertension and tachycardia is the mainstay treatment principle to reduce shear stress on the arterial wall, which may lead to rupture [12]. In ICH and neurocritical care, studies of RPP have been limited. Two retrospective studies evaluated the association of RPP with in-hospital mortality following severe traumatic brain injury and aneurysmal subarachnoid hemorrhage, respectively [13,14]. While RPP was associated with early mortality, the mechanism by which mortality is increased is unknown.

We therefore analyzed the Field Administration of Stroke Therapy-Magnesium (FAST-MAG) study data to investigate the relationship between RPP and HE in the hyperacute period that includes the pre-hospital setting. The FAST-MAG trial was a randomized, double-blind, placebo-controlled trial for acute stroke within 2 h of onset to study the efficacy of neuroprotective magnesium sulfate therapy initiated by paramedics in the field [15]. The design of the trial provided an opportunity to evaluate ICH patients in an earlier timeframe after symptom onset compared to most clinical trials [7,8]. We hypothesized that RPP measurements in the hyperacute and acute periods were associated with both increased initial hematoma volume and subsequent HE in a population of patients with acute spontaneous ICH. The results of this study are anticipated to improve our understanding of the clinical factors that contribute to early HE and secondary brain injury, investigating a new potential physiologic target for treatment and research.

## 2. Materials and Methods

### 2.1. Study Design and Data Source

This retrospective cohort study investigated the association between RPP and early hematoma characteristics, including HE, in a population of acute non-traumatic ICH. For this study, we performed a secondary analysis using data from the NIH-funded multicenter FAST-MAG trial. The details of the FAST-MAG trial protocol, baseline characteristics, and main trial results have been previously described [15]. In brief, the study enrolled 1700 patients with 1:1 randomization to pre-hospital neuroprotective agent magnesium sulfate or placebo within 2 h of stroke symptom onset. The clinical trial included patients identified from 315 paramedic-staffed ambulances and 60 receiving hospital sites in Los Angeles County and Orange County, California. Participants were enrolled between January 2005 and March 2013. Informed consent and institutional review board approval were not required. This secondary analysis utilized existing de-identified data, documents, and records in an anonymous manner such that study participants from the original study were not identified.

### 2.2. Study Population

The original FAST-MAG study inclusion criteria selected participants aged 40–95 years old, suspected stroke identified by the modified Los Angeles Pre-hospital Stroke Screen, last known well time within 2 h of treatment initiation, and deficit present for >15 min. Exclusion criteria included SBP <90 or >220 mmHg, major head trauma in the last 24 h, rapidly improving deficit, coma, severe respiratory distress, pre-existing systemic disease that would confound outcome evaluations, recent stroke in the last 30 days, or lack of a legal representative if the patient was unable to give informed consent.

**Figure 1:**
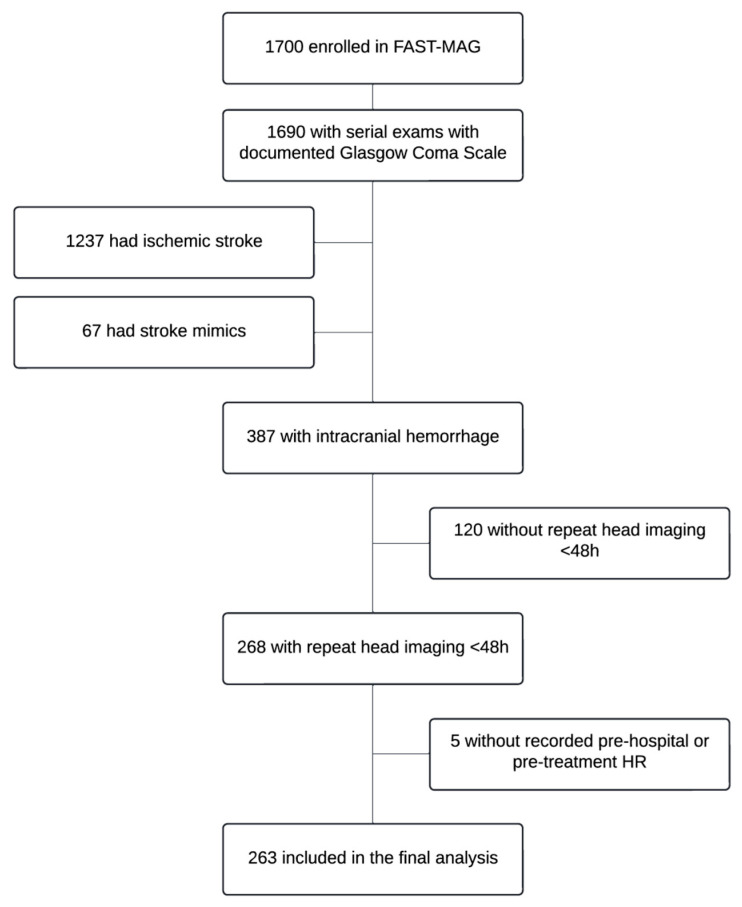
Flow diagram of secondary dataset analysis from the FAST-MAG trial. HR = heart rate.

For our secondary analysis, 263 patients from the FAST-MAG trial with a primary diagnosis of spontaneous ICH were included (Figure 1). All subjects underwent brain imaging upon hospital arrival to confirm the primary diagnosis. For patients who had ICH diagnosed, serial surveillance neuroimaging within 48 h from the first scan was obtained per study protocol. Five subjects with ICH but without HE were excluded due to the absence of a recorded pre-hospital or pre-treatment HR. The complete original study methodologies are described in the published FAST-MAG protocols [16].

### 2.3. Outcome Variables

The primary outcome of interest was initial hematoma volume by quintiles of hyperacute RPP. The secondary outcome of interest was the occurrence of HE in the same RPP quintiles. HE was defined by volume expansion of >6 mL or >33% from baseline volume on repeat neuroimaging performed within 48 h of the first scan. In the original FAST-MAG dataset, the initial hematoma volume was calculated using the ABC/2 method. All images were reviewed and interpreted by an experienced neuroradiologist.

The hemodynamic variables included in our analysis were HR, SBP, and diastolic blood pressure (DBP), measured in both the pre-hospital and pre-treatment windows, stratified by HE occurrence. For each patient case, RPP was calculated as the product of HR x SBP. The pre-treatment RPP value used for analysis was the average of the calculated RPP obtained in both pre-hospital and pre-treatment windows. Pre-hospital values were obtained in the field. Pre-treatment values were defined as vitals upon emergency room arrival and prior to initiation of antihypertensive drug treatment. The hyperacute period was defined as the combined pre-hospital and pre-treatment windows.

### 2.4. Statistical Analysis

Patient demographics and clinical characteristics were reported as a mean with standard deviation (SD) for continuous variables, median with interquartile range (IQR) for numeric discrete variables, and counts with frequency for categorical variables. For continuous hemodynamic variables, the average value in the hyperacute period was calculated and used for analysis. Pre-treatment averages were obtained from a single data point in 21 cases due to the absence of additional measurements. Test for differences between those with and without HE was performed using t-tests or Wilcoxon rank sum tests for numeric variables and chi-square or Fisher’s exact tests for categorical variables. Results are presented stratified by HE status. We further tested demographics and clinical characteristics stratified by RPP quintile using one-way ANOVA with Bonferroni-adjusted pairwise comparisons for numeric variables and Pearson’s or Fisher’s exact test for categorical variables.

To assess the degree to which RPP affects HE, we used multivariable logistic regression to obtain odds ratios (ORs) and 95% confidence intervals (CIs). Covariates of interest included: antithrombotic/anticoagulant use (dichotomized as yes vs. no), antiplatelet use (yes/no), initial hematoma volume (mL), female sex, hypertension, hyperlipidemia, diabetes, coronary artery disease (CAD), atrial fibrillation, tobacco use (yes/no), age, Los Angeles Motor Scale (LAMS) score (dichotomized as 0–3 vs. 4–5), and average DBP (mmHg). RPP was modeled into ranked quintiles to align with previous literature [17]. To develop the model, we evaluated all variables listed above in an iterative manner, as well as any variable with a univariate *p*-value < 0.25. Only variables that were significantly associated with the outcome or were confounders were included in the final model. A confounder was defined as any variable that, when added to the model, altered the effect size between RPP and HE by >15%. Model assumptions and fit were assessed using the Hosmer–Lemeshow goodness-of-fit test and inspection of plots, leverage, and deviance. Missing data on covariates were excluded. All analyses were conducted using Stata 18.0 (StataCorp, College Station, TX, USA). A *p*-value of <0.05 was considered statistically significant.

Post hoc, we calculated the effect size detectable with the available 268 patients, of whom 116 (44%) developed HE. We assumed 80% power, a two-sided type I error rate of 5%, a baseline 37% probability of HE under the null [18], and an R^2^ for other covariates of 0.20. Given these assumptions, we were able to detect an odds ratio (OR) of 1.51. Power calculations were conducted using G*Power 3.1.

## 3. Results

Among the 387 patients with acute ICH, a total of 268 (69.2%) had at least one repeat neuroimaging within 48 h. A total of 263 (67.9%) patients had data points at the timepoints of interest and were included in the final analysis (Figure 1). The median age of all patients was 65 years old, with 66.8% of the population being male and primarily white and non-Hispanic. HE occurred in 116 (44%) individuals. Table 1 shows baseline characteristics by quintile of calculated RPP. Patients across different quintiles of RPP generally had similar characteristics; however, those in lower RPP quintiles were older and more likely to have hyperlipidemia and CAD. On average, the initial hematoma volume was largest in Quintiles 1–2 compared to Quintiles 3–5, but the imbalances did not reach statistical significance. There was also no statistically significant imbalance in antiplatelet or anticoagulant drug use across quintiles.

**Figure 2:**
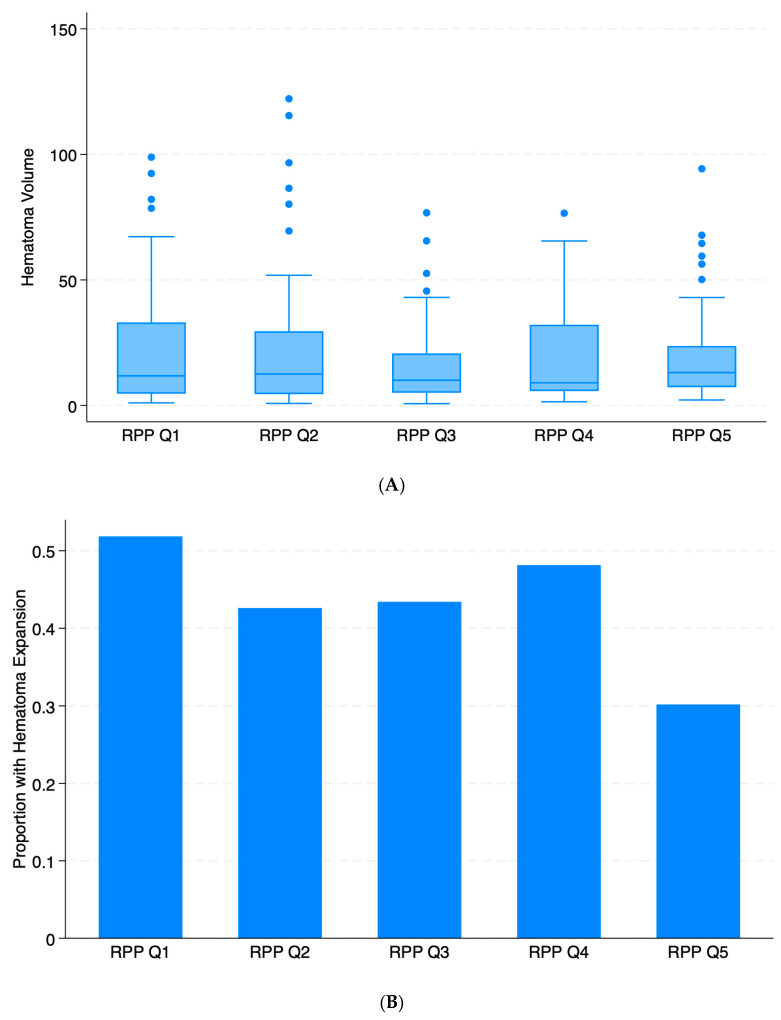
Primary and secondary outcomes by quintiles of rate-pressure product. (**A**) Distribution of initial hematoma volume by quintiles of rate-pressure product is shown in the box-plot diagram. (**B**) Proportions of subjects with hematoma expansion by quintiles of rate-pressure product. RPP = rate-pressure product; Q1 = Quintile 1; Q2 = Quintile 2; Q3 = Quintile 3; Q4 = Quintile 4; Q5 = Quintile 5.

Figure 2A shows the initial hematoma volume by RPP quintile. Overall, there was high variability in initial hematoma volume in each quintile, and no trend increase or decrease was observed across all quintiles. Figure 2B displays the occurrence of HE by RPP quintiles. The proportion of patients with HE was greatest in Quintile 1 and was lowest in Quintile 5, but no discernible pattern or trend was observed across all quintiles.

Using Quintile 1 as the comparison, multivariate logistic regression demonstrated a smaller adjusted OR for HE in Quintile 5 (OR 0.38, CI 0.17–0.86, *p* = 0.020) after controlling for female sex and anticoagulation use. The adjusted ORs of HE were significantly increased by anticoagulation use and female gender (Table 2). Quintile 2–4 showed ORs < 1 for HE, but the results did not demonstrate statistical significance.

## 4. Discussion

In our secondary analysis of the prospective FAST-MAG randomized controlled trial (RCT), increased RPP in the hyperacute period prior to BP treatment in the emergency department was not positively associated with initial hematoma volume or occurrence of early HE in a population of spontaneous ICH. This result does not support our hypothesis that higher RPP is associated with worse hematoma characteristics due to increased shear stress from elevated SBP in combination with elevated HR. This is the first known report of the association between hyperacute RPP and early hematoma characteristics.

To test our hypothesis, we used data from the well-designed FAST-MAG RCT, which longitudinally captured hyperacute vital signs and neuroimaging in a well-defined ICH population. Despite the strengths of the trial, imbalances in important covariates within our cohort between quintile study groups may have contributed to our negative result. Patients in RPP Quintile 1 for RPP were older, more likely to have hyperlipidemia and coronary artery disease, and had a larger initial hematoma volume. Antiplatelet drug use was also highest in Quintile 1 and lowest in Quintile 5, with no anticoagulant use among individuals in Quintile 5. Although we attempted to account for confounders and effect modifiers in our multivariate logistic regression, these baseline imbalances would bias our findings towards the null hypothesis. Furthermore, although our study is a reasonable representation of spontaneous ICH patients, selection biases exist based on the criteria of the original FAST-MAG study. Of special importance, the inclusion criteria of age 40–95 years of age and exclusion criteria of coma or SBP < 90 or > 220 mmHg may have skewed the study towards an older population and excluded the most severe ICH cases, including those with extremely high and low RPP, who may be at the highest risk of HE.

The potential effect of magnesium exposure in our cohort is unclear. The treatment and placebo groups were well-balanced in the original RCT, suggesting no differential bias in our cohort [15]. Magnesium could systematically lower SBP and HR due to its vasodilatory effects [19]. A recent secondary analysis of the FAST-MAG ICH subgroup by Liotta et al. showed that a higher level of serum magnesium was associated with less HE, suggesting a hemostatic effect that could lead to a bias toward the null hypothesis regarding the association between RPP and HE [20].

In multivariable analysis, we found that the highest RPP quintile demonstrated a statistically significant decrease in OR for HE, but the clinical significance of this finding is unclear, as no clear trend across the remaining quintiles was observed. Although we hypothesized that higher RPP would linearly correlate with HE due to increased shear stress, our results suggest a more complex, non-linear relation with disproportionate impact on HE among very high RPP values. Beyond confounding from antithrombotic use, this paradoxical observation may reflect confounding by indication, where patients presenting with extreme hemodynamic values may have had concurrent medical emergencies that necessitated more aggressive pre-hospital stabilization. Such prioritization with resuscitation may have inadvertently lowered the risk of hematoma growth. Therefore, our findings should be considered as exploratory and hypothesis-generating.

The lack of a linear association between high RPP and ultra-early hematoma size and growth may indicate that, rather than hemodynamics, biologic factors and baseline patient characteristics, such as age, pre-existing cardiovascular disease, and antithrombotic drug use, are more potent determinants in the development of ICH and in outcome prediction. Prior studies have demonstrated that age is significantly associated with death and disability following ICH [21], while antithrombotic use similarly predicts mortality and poor neurologic outcome [22]. Although these factors are not feasibly modifiable acutely after ICH onset, this highlights the importance of primary and secondary prevention of cardiovascular disease, the appropriate prescription of antithrombotic drugs in those at high risk of ICH, and promotes further study on reversing drug-induced coagulopathies to prevent HE and secondary brain injury [23].

Based on current medical literature, the true influence of early hemodynamics on ICH outcomes remains uncertain. Despite showing a reduction in HE, landmark clinical trials such as INTERACT-2 and ATACH-2 failed to demonstrate a consistent clinical benefit of intensive BP control in the first few days after ICH [7,8]. More recently, a meta-analysis by Moullaali et al. indicated that a lower BP within 7 days of ICH onset reduced hematoma growth but did not improve functional recovery [24]. Although the most recent INTERACT-3 trial showed that a care bundle including intensive blood pressure management was associated with better functional outcomes, the beneficial effect of protocolized care is at risk of bias in determining causation [25]. Research by Divani et al. further suggests that large reductions in SBP early after ICH may actually be harmful [26]. Our study specifically investigated the influence of SBP and HR in the ultra-early phase after ICH onset, when patients are at maximal risk for HE, and did not find a positive association. In addition, we recently failed to show an association between blood pressure variability (BPV) and hematoma growth in this same dataset [17]. Together, these results suggest that hemodynamic variables such as BP, HR, RPP, and BPV may not be robust contributors to long-term functional outcomes, and their role in goal-directed therapy following ICH remains uncertain.

The significance of RPP in ICH and other cerebrovascular diseases should be more extensively studied. Although primarily established as a parameter for myocardial oxygenation and workload in cardiac disease [27,28,29], RPP is considered a novel indirect marker for autonomic dysregulation after acquired brain injury. Krishnamoorthy et al. showed that in patients with severe traumatic brain injury, both elevated and depressed RPPs were associated with increased mortality [13]. Zhao et al. identified a similar biphasic effect of RPP in association with in-hospital mortality in patients with aneurysmal subarachnoid hemorrhage [14]. Deviations in RPP, at either extreme, may be indicative of impaired cerebral autoregulation, increasing the risk of secondary brain injury from either hypertensive crisis or cerebral ischemia due to suboptimal perfusion. Indeed, in a series of studies by Diedler et al., cerebrovascular dysregulation was commonly observed following large spontaneous ICH and was associated with poor clinical outcomes [30,31]. Given that acute brain injury most often triggers a sympathetic surge that may increase cardiac workload, the association between RPP and cardiac function in the context of acquired brain injuries, including ICH, warrants further research [32].

Other limitations should be discussed. The retrospective design of this secondary database analysis limits the ability to explore causal relationships. With a retrospective cohort, we were unable to account for all important variables that might influence RPP and hematoma growth. Prescribed medications like antihypertensive beta-blockade can blunt the sympathetic response after ICH, potentially reducing observed RPP. Additionally, the specific hourly timestamps for the follow-up neuroimaging were unavailable for analysis. This heterogeneity in follow-up imaging could affect the detection of HE, as delayed HE may be missed. Our study is also limited by the available measurement frequency of RPP. Specifically, the pre-treatment RPP of 21 patients was derived from a single measurement. While this could potentially lead to exposure misclassification, these patients represented a small fraction of our study population. Moreover, ICH location, appearance, or the presence of intraventricular hemorrhage, which are crucial variables likely to impact hematoma characteristics and behavior, were not recorded in the original FAST-MAG trial and were therefore not factored into our study [33,34]. Beyond HR and BP, other physiologic confounders may have been present. Lastly, while our post hoc analysis using a quintile-based approach helps to identify non-linearity and improves interpretability, the categorical approach may result in information loss. A larger study population would be required to maintain sufficient statistical power for more complex analyses, enabling the detection of smaller effect sizes. Given these limitations, additional studies are recommended to better control for clinically relevant variables we were unable to measure.

## 5. Conclusions

In this secondary analysis of the FAST-MAG dataset, elevated RPP (SBP × HR) was not associated with increased initial hematoma volume or subsequent HE in the hyperacute period after spontaneous ICH. This is the first study to investigate the relationship between this non-invasive hemodynamic variable and hematoma characteristics. Future research is recommended to better elucidate the clinical utility of RPP as a biomarker of autoregulation and cardiac workload, particularly in a more severely ill ICH population while controlling for key pharmacologic and physiologic prognostic factors.

There is an increasing clinical imperative for timely, ultra-early intervention in ICH [35]. Integrating RPP and radiographic phenotypes as a multi-modal approach can help delineate the mechanistic links between the hemodynamics of ICH and hematoma behavior, thereby informing ultra-early clinical decision-making. Ultimately, understanding how RPP and early hematoma characteristics are influenced by patient-level factors such as age, gender, co-morbidities, medication use, and severity of illness may yield better physiologic targets to tailor ICH management. With further clinical validation, RPP might serve as a novel tool to risk-stratify ICH progression and subsequent medical complications, facilitating successful bundled care interventions.

## Figures and Tables

**Table 1:** Demographic and Clinical Characteristics of the Study Population, Stratified According to Quintiles of Rate-Pressure Product. RPP = Rate-pressure product; SD = Standard deviation; LAMS = Los Angeles motor scale; SBP = systolic blood pressure; DBP = diastolic blood pressure. BPM = Beats per minute.

	RPP Quintile 1 (N = 54)	RPP Quintile 2 (N = 54)	RPP Quintile 3 (N = 53)	RPP Quintile 4 (N = 54)	RPP Quintile 5 (N = 53)	
Variable ^1^	Mean ± SD or Count (%)	Mean ± SD or Count (%)	Mean ± SD or Count (%)	Mean ± SD or Count (%)	Mean ± SD or Count (%)	*p*-Value ^2^
Demographics						
Age	68.4 ± 14.2	67.8 ± 13.4	62.7 ± 12.6	61.4 ± 12.3	63.6 ± 11.5	0.014
Sex Male Female	41 (75.9) 13 (24.1)	29 (53.7) 25 (46.3)	40 (75.5) 13 (24.3)	38 (70.4) 16 (29.6)	31 (58.5) 22 (41.5)	0.041
Ethnicity Not Hispanic/Latino Hispanic/Latino	39 (72.2) 15 (27.8)	42 (77.8) 12 (22.2)	35 (66.0) 18 (34.0)	29 (53.7) 25 (46.3)	34 (64.2) 19 (35.9)	0.091
Race White Black Asian Native American/Native Hawaiian/ Alaska Native	48 (88.9) 3 (5.6) 3 (5.6) 0 (0)	42 (77.8) 3 (5.6) 8 (14.8) 1 (1.9)	40 (75.5) 5 (9.4) 5 (9.4) 3 (5.7)	42 (77.8) 6 (11.1) 5 (9.3) 1 (1.9)	40 (75.5) 6 (11.3) 7 (13.2) 0 (0)	0.569
Medical History						
Hypertension No Yes	14 (25.9) 40 (74.1)	9 (16.7) 45 (83.3)	13 (24.5) 40 (75.5)	7 (13.0) 47 (87.0)	14 (26.4) 39 (73.6)	0.310
Diabetes No Yes	43 (79.6) 11 (20.4)	48 (88.9) 6 (11.1)	47 (88.7) 6 (11.3)	42 (77.8) 12 (22.2)	40 (75.5) 13 (24.5)	0.219
Hyperlipidemia No Yes	28 (51.9) 26 (48.2)	28 (51.9) 26 (48.2)	40 (75.5) 13 (24.5)	33 (61.1) 21 (38.9)	38 (71.7) 15 (28.3)	0.027
Atrial Fibrillation No Yes	49 (90.7) 5 (9.3)	50 (92.6) 4 (7.4)	48 (90.6) 5 (9.4)	49 (90.7) 5 (9.3)	51 (96.2) 2 (3.8)	0.753
Coronary Artery Disease No Yes	40 (74.1) 14 (15.9)	49 (90.7) 5 (9.3)	51 (96.2) 2 (3.8)	47 (87.0) 7 (13.0)	51 (96.2) 2 (3.8)	0.002
Antiplatelet Use No Yes	32 (59.3) 22 (40.7)	37 (68.5) 17 (31.5)	38 (71.7) 15 (28.3)	34 (63.0) 28 (37.0)	40 (75.5) 13 (24.5)	0.384
Anticoagulant Use No Yes	51 (94.4) 3 (5.6)	49 (90.7) 5 (9.3)	48 (90.6) 5 (9.4)	51 (94.4) 3 (5.6)	53 (100) 0 (0)	0.159
Antiplatelet or Anticoagulant Use No Yes	29 (53.7) 25 (46.3)	33 (61.1) 21 (38.9)	35 (66.0) 18 (34.0)	31 (57.4) 23 (42.6)	40 (75.5) 13 (24.5)	0.163
Tobacco Use No Yes	50 (92.6) 4 (7.4)	46 (85.2) 8 (14.8)	42 (79.3) 11 (20.8)	46 (85.2) 8 (14.8)	42 (79.3) 11 (20.8)	0.266
Arrival Condition						
Modified Rankin Scale 0 2 3	49 (90.7) 3 (5.6) 2 (3.7)	50 (92.6) 4 (7.4) 0 (0)	50 (94.3) 2 (3.8) 1 (1.9)	49 (90.7) 3 (5.6) 2 (3.7)	50 (94.3) 2 (3.8) 1 (1.9)	0.952
LAMS <4 ≥4	14 (25.9) 40 (74.1)	13 (24.1) 41 (75.9)	10 (18.9) 43 (81.1)	11 (20.4) 43 (79.6)	4 (7.6) 49 (92.5)	0.134
Time from Symptom Onset to First Brain Scan (hours)	1.5 ± 0.6	1.4 ± 0.4	1.3 ± 2.0	1.3 ± 0.5	1.4 ± 1.0	0.970
Initial Hematoma Volume (ml)	23.1 ± 25.4	24.6 ± 29.4	16.6 ± 16.8	18.7 ± 17.9	19.6 ± 19.3	0.328
Pulse (BPM)	66.7 ± 9.2	71.8 ± 9.2	82.6 ± 10.4 ^3,4^	85.2 ± 11.5 ^3,4^	95.7 ± 13.0 ^3,4,5,6^	<0.001
SBP (mmHg)	148.4 ± 25.6	172.1 ± 24.8	174.9 ± 29.1 ^3^	188.1 ± 28.4 ^3,4^	199.1 ± 25.9 ^3,4,5^	<0.001
DBP (mmHg)	78.4 ±14.8	93.8 ± 17.5	97.4 ± 19.1 ^3^	107.8 ± 20.4 ^3,4,5^	109.0 ± 22.3 ^3,4,5^	<0.001
RPP (BPM x mmHg)	10,245.0 ± 1170.1	12,731.9 ± 504.3	140,732.4 ± 449.1	15,906.9 ± 681.0	19,089.7 ± 1728.2	<0.001

^1^ Numeric variables are presented as mean and standard deviation, while categorical variables are presented as count (frequency). Chi-square or Fisher’s Exact tests were used for categorical variables. ^2^ *p*-values were obtained using one-way ANOVA with Bonferroni correction for numeric variables and Pearson’s chi-square or Fisher’s exact test. ^3^ Statistically significantly different from Quintile 1 at *p* = 0.05, Bonferroni-adjusted. ^4^ Statistically significantly different from Quintile 2 at *p* = 0.05, Bonferroni-adjusted. ^5^ Statistically significantly different from Quintile 3 at *p* = 0.05, Bonferroni-adjusted. ^6^ Statistically significantly different from Quintile 4 at *p* = 0.05, Bonferroni-adjusted.

**Table 2:** Multivariate Regression Model of the Association Between Rate-Pressure Product and Hematoma Expansion. OR = odds ratio; CI = confidence interval; RPP = rate-pressure product.

Variables	OR	95% CI	*p*-Value
Anticoagulation use	3.02	1.0–9.16	0.051
Female sex	1.67	0.98–2.86	0.061
RPP Quintiles			
1	(1.00)	-	-
2	0.58	0.27–1.28	0.178
3	0.67	0.31–1.46	0.318
4	0.85	0.39–1.80	0.652
5	0.38	0.17–0.86	0.020

## Data Availability

The data that support the findings of this study are openly available and can be accessed by visiting the study’s record on ClinicalTrials.gov (NCT00059332).

## Abbreviations

The following abbreviations are used in this manuscript:

ICH	Intracerebral hemorrhage
HE	Hematoma expansion
RPP	Rate-pressure product
FAST-MAG	Field administration of stroke therapy-magnesium
SBP	Systolic blood pressure
BP	Blood pressure
INTERACT	Intensive blood pressure reduction in acute cerebral hemorrhage trials
ATACH	Antihypertensive treatment of acute cerebral hemorrhage
HR	Heart rate
DBP	Diastolic blood pressure
SD	Standard deviation
IQR	Interquartile range
OR	Odds ratio
CI	Confidence intervals
CAD	Coronary artery disease
LAMS	Los Angeles motor scale
RCT	Randomized controlled trial
BPV	Blood pressure variability

## References

[1] Wang S., Zou X.-L., Wu L.-X., Zhou H.-F., Xiao L., Yao T., Zhang Y., Ma J., Zeng Y., Zhang L. (2022). Epidemiology of intracerebral hemorrhage: A systematic review and meta-analysis. Front. Neurol..

[2] Feigin V.L., Forouzanfar M.H., Krishnamurthi R., A Mensah G., Connor M., Bennett D.A., Moran A.E., Sacco R.L., Anderson L., Truelsen T. (2014). Global and regional burden of stroke during 1990–2010: Findings from the Global Burden of Disease Study 2010. Lancet.

[3] The Editorial Committee for Japan Stroke Data Bank 2021 in the National Cerebral and Cardiovascular Center (2021). Japan Stroke Data Bank 2021.

[4] Fernando S.M., Qureshi D., Talarico R., Tanuseputro P., Dowlatshahi D., Sood M.M., Smith E.E., Hill M.D., McCredie V.A., Scales D.C. (2021). Intracerebral Hemorrhage Incidence, Mortality, and Association with Oral Anticoagulation Use: A Population Study. Stroke.

[5] Qureshi A.I., Ezzeddine M.A., Nasar A., Suri M.F.K., Kirmani J.F., Hussein H.M., Divani A.A., Reddi A.S. (2007). Prevalence of elevated blood pressure in 563,704 adult patients with stroke presenting to the ED in the United States. Am. J. Emerg. Med..

[6] Larsen K.T., Selseth M.N., Jahr S.H., Hillestad V., Koubaa N., Sandset E.C., Rønning O.M., Kristoffersen E.S. (2022). Prehospital Blood Pressure and Clinical and Radiological Outcomes in Acute Spontaneous Intracerebral Hemorrhage. Stroke.

[7] Qureshi A.I., Palesch Y.Y., Barsan W.G., Hanley D.F., Hsu C.Y., Martin R.L., Moy C.S., Silbergleit R., Steiner T., Suarez J.I. (2016). Intensive Blood-Pressure Lowering in Patients with Acute Cerebral Hemorrhage. N. Engl. J. Med..

[8] Anderson C.S., Heeley E., Huang Y., Wang J., Stapf C., Delcourt C., Lindley R., Robinson T., Lavados P., Neal B. (2013). Rapid blood-pressure lowering in patients with acute intracerebral hemorrhage. N. Engl. J. Med..

[9] Hagel J.A., Sperotto F., Laussen P.C., Salvin J.W., Bachu A., Kheir J.N. (2021). Shock Index, Coronary Perfusion Pressure, and Rate Pressure Product As Predictors of Adverse Outcome After Pediatric Cardiac Surgery. Pediatr. Crit. Care Med..

[10] Kiviniemi A.M., Kenttä T.V., Lepojärvi S., Perkiömäki J.S., Piira O.-P., Ukkola O., Huikuri H.V., Junttila M.J., Tulppo M.P. (2019). Recovery of rate-pressure product and cardiac mortality in coronary artery disease patients with type 2 diabetes. Diabetes Res. Clin. Pract..

[11] Verma A.K., Sun J., Hernandez A., Teerlink J.R., Schulte P.J., Ezekowitz J., Voors A., Starling R., Armstrong P., O’Conner C.M. (2018). Rate pressure product and the components of heart rate and systolic blood pressure in hospitalized heart failure patients with preserved ejection fraction: Insights from ASCEND-HF. Clin. Cardiol..

[12] Isselbacher E.M., Preventza O., Black J.H., Augoustides J.G., Beck A.W., Bolen M.A., Braverman A.C., Bray B.E., Brown-Zimmerman M.M., Chen E.P. (2022). 2022 ACC/AHA Guideline for the Diagnosis and Management of Aortic Disease: A Report of the American Heart Association/American College of Cardiology Joint Committee on Clinical Practice Guidelines. Circulation.

[13] Krishnamoorthy V., Vavilala M.S., Chaikittisilpa N., Rivara F.P., Temkin N.R., Lele A.V., Gibbons E.F., Rowhani-Rahbar A. (2018). Association of Early Myocardial Workload and Mortality Following Severe Traumatic Brain Injury. Crit. Care Med..

[14] Zhao J., Zhang S., Ma J., Shi G., Zhou J. (2022). Admission rate-pressure product as an early predictor for in-hospital mortality after aneurysmal subarachnoid hemorrhage. Neurosurg. Rev..

[15] Saver J.L., Starkman S., Eckstein M., Stratton S.J., Pratt F.D., Hamilton S., Conwit R., Liebeskind D.S., Sung G., Kramer I. (2015). Prehospital use of magnesium sulfate as neuroprotection in acute stroke. N. Engl. J. Med..

[16] Saver J.L., Starkman S., Eckstein M., Stratton S., Pratt F., Hamilton S., Conwit R., Liebeskind D.S., Sung G., Sanossian N. (2014). Methodology of the Field Administration of Stroke Therapy—Magnesium (FAST-MAG) phase 3 trial: Part 1—Rationale and general methods. Int. J. Stroke.

[17] Oh D.M., Shkirkova K., Poblete R.A., Chung P.-W., Saver J.L., Starkman S., Liebeskind D.S., Hamilton S., Wilson M., Sanossian N. (2023). Association Between Hyperacute Blood Pressure Variability and Hematoma Expansion After Intracerebral Hemorrhage: Secondary Analysis of the FAST-MAG Database. Neurocrit. Care.

[18] Jauch E.C., Lindsell C., Broderick J., Fagan S.C., Tilley B.C., Levine S.R. (2006). Association of serial biochemical markers with acute ischemic stroke: The National Institute of Neurological Disorders and Stroke recombinant tissue plasminogen activator Stroke Study. Stroke.

[19] Houston M. (2011). The role of magnesium in hypertension and cardiovascular disease. J. Clin. Hypertens..

[20] Liotta E.M., Maas M.B., Prabhakaran S., Shkirkova K., Sanossian N., Liebeskind D.S., Sharma L., Stratton S., Conwit R., Saver J.L. (2024). Magnesium and Hematoma Expansion in Intracerebral Hemorrhage: A FAST-MAG Randomized Trial Analysis. Stroke.

[21] Rådholm K., Arima H., Lindley R.I., Wang J., Tzourio C., Robinson T., Heeley E., Anderson C.S., Chalmers J. (2015). Older age is a strong predictor for poor outcome in intracerebral haemorrhage: The INTERACT2 study. Age Ageing.

[22] Apostolaki-Hansson T., Ullberg T., Pihlsgård M., Norrving B., Petersson J. (2021). Prognosis of Intracerebral Hemorrhage Related to Antithrombotic Use: An Observational Study From the Swedish Stroke Register (Riksstroke). Stroke.

[23] Sawalha K., Kamdar H.A., Gullo T., Okere S., Hamed M., Hinduja A., Hussein O. (2022). Cardiovascular Predictors of Intracerebral Hematoma Expansion. J. Stroke Cerebrovasc. Dis..

[24] Moullaali T.J., Wang X., Sandset E.C., Woodhouse L.J., Law Z.K., Arima H., Butcher K.S., Chalmers J., Delcourt C., Edwards L. (2022). Early lowering of blood pressure after acute intracerebral haemorrhage: A systematic review and meta-analysis of individual patient data. J. Neurol. Neurosurg. Psychiatry.

[25] Ma L., Hu X., Song L., Chen X., Ouyang M., Billot L., Li Q., Malavera A., de Silva A., Thang N.H. (2023). The third Intensive Care Bundle with Blood Pressure Reduction in Acute Cerebral Haemorrhage Trial (INTERACT3): An international, stepped wedge cluster randomised controlled trial. Lancet.

[26] Divani A.A., Liu X., Petersen A., Lattanzi S., Anderson C.S., Ziai W., Torbey M.T., Moullaali T.J., James M.L., Jafarli A. (2020). The Magnitude of Blood Pressure Reduction Predicts Poor In-Hospital Outcome in Acute Intracerebral Hemorrhage. Neurocrit. Care.

[27] Kal J.E., Van Wezel H.B., Vergroesen I. (1999). A critical appraisal of the rate pressure product as index of myocardial oxygen consumption for the study of metabolic coronary flow regulation. Int. J. Cardiol..

[28] Fletcher G.F., Ades P.A., Kligfield P., Arena R., Balady G.J., Bittner V.A., Coke L.A., Fleg J.L., Forman D.E., Gerber T.C. (2013). Exercise standards for testing and training: A scientific statement from the American Heart Association. Circulation.

[29] Zhou J., Li Y.J., Zhou X.D., Wang L.J. (2024). Rate-Pressure Product is a Novel Predictor for Short- and Long-Term Mortality in Patients with Acute Coronary Syndrome Undergoing Primary PCI/Immediate Invasive Strategy. Clin. Interv. Aging.

[30] Diedler J., Sykora M., Rupp A., Poli S., Karpel-Massler G., Sakowitz O., Steiner T. (2009). Impaired cerebral vasomotor activity in spontaneous intracerebral hemorrhage. Stroke.

[31] Diedler J., Santos E., Poli S., Sykora M. (2014). Optimal cerebral perfusion pressure in patients with intracerebral hemorrhage: An observational case series. Crit. Care.

[32] Krishnamoorthy V., Mackensen G.B., Gibbons E.F., Vavilala M.S. (2016). Cardiac Dysfunction After Neurologic Injury: What Do We Know and Where Are We Going?. Chest.

[33] Jolink W.M.T., Wiegertjes K., Rinkel G.J.E., Algra A., de Leeuw F.E., Klijn C.J.M. (2020). Location-specific risk factors for intracerebral hemorrhage: Systematic review and meta-analysis. Neurology.

[34] Chung G.H., Goo J.H., Kwak H.S., Hwang S.B. (2022). The comprehensive comparison of imaging sign from CT angiography and noncontrast CT for predicting intracranial hemorrhage expansion: A comparative study. Medicine.

[35] Li Q., Yakhkind A., Alexandrov A.W., Anderson C.S., Dowlatshahi D., Frontera J.A., Hemphill J.C., Ganti L., Kellner C., May C. (2024). Code ICH: A Call to Action. Stroke.

